# Protein restriction and branched‐chain amino acid restriction promote geroprotective shifts in metabolism

**DOI:** 10.1111/acel.13626

**Published:** 2022-05-08

**Authors:** Michaela E. Trautman, Nicole E. Richardson, Dudley W. Lamming

**Affiliations:** ^1^ Department of Medicine University of Wisconsin‐Madison Madison Wisconsin USA; ^2^ William S. Middleton Memorial Veterans Hospital Madison Wisconsin USA; ^3^ Interdepartmental Graduate Program in Nutritional Sciences University of Wisconsin‐Madison Madison Wisconsin USA; ^4^ Endocrinology and Reproductive Physiology Graduate Training Program University of Wisconsin‐Madison Madison Wisconsin USA

**Keywords:** amino acids, branched‐chain amino acids, FGF21, isoleucine, lifespan, mTOR

## Abstract

The proportion of humans suffering from age‐related diseases is increasing around the world, and creative solutions are needed to promote healthy longevity. Recent work has clearly shown that a calorie is not just a calorie—and that low protein diets are associated with reduced mortality in humans and promote metabolic health and extended lifespan in rodents. Many of the benefits of protein restriction on metabolism and aging are the result of decreased consumption of the three branched‐chain amino acids (BCAAs), leucine, isoleucine, and valine. Here, we discuss the emerging evidence that BCAAs are critical modulators of healthy metabolism and longevity in rodents and humans, as well as the physiological and molecular mechanisms that may drive the benefits of BCAA restriction. Our results illustrate that protein quality—the specific composition of dietary protein—may be a previously unappreciated driver of metabolic dysfunction and that reducing dietary BCAAs may be a promising new approach to delay and prevent diseases of aging.

## Graphical abstract

Creative solutions are needed to combat increasing rates of metabolic disease and promote healthy aging. This review summarizes the latest research on protein and branched‐chain amino acid restriction as interventions to alter metabolism to extend lifespan and reduce frailty in model organisms and humans. The authors discuss the potential mechanisms underpinning these dietary interventions and postulate future directions of the field, which may include personalized nutrition therapy.

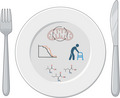

## 1 INTRODUCTION

Around the globe, human life expectancy increased by almost 20 years between 1950 and 2017 (Collaborators, 2018). Despite the effect of the global COVID‐19 pandemic, which has caused life expectancy in the United States to slightly decline (Arias et al., 2021), advances in medicine have shifted population demographics, and humans older than 65 now represent the fastest growing age group worldwide (United Nations, 2019). As a result, the portion of deaths attributed to noncommunicable diseases, such as age‐related diseases, has risen and will continue to rise (Foreman et al., 2018).

Though human life expectancy has largely increased, the prevalence of obesity and related disorders has grown rapidly, threatening the quality and duration of healthy years for an ever‐expanding aged population. Obesity is more than tripled in men and doubled in women from 1975 to 2014, and 43% of American adults aged 40–59 are now obese (Collaboration, 2016). Obesity is increasingly impacting younger individuals, with about 40% of children now overweight or obese, predisposing them to chronic diseases at younger ages and lifelong challenges maintaining a healthy body weight (*Dietary Guidelines for Americans*, *2020*–*2025*, 2020). This increase comes despite the fact that the Healthy Eating Index, a score of diet quality, has not shown significant declines since the early 2000s and in fact may be higher in recent years (*Dietary Guidelines for Americans*, *2020*–*2025*, 2020). This suggests that perhaps another factor besides declining food habits is at play. Creative solutions are needed to combat these conditions and preserve quality of life, as obesity is a risk factor for many age‐related diseases, including metabolic, cardiovascular, neurodegenerative, and musculoskeletal conditions, depression, and some cancers (Bluher, 2019). One of these solutions may be targeting aging itself to promote healthspan rather than attempt to treat the myriad of age‐related diseases present in the elderly (Partridge, 2014).

## 2 DIETARY INTERVENTIONS CAN PROMOTE HEALTHY AGING

Calorie restriction (CR), defined as a decrease in caloric intake without malnutrition, is often referred to as the “gold standard” of nutritional interventions, for its potent ability to preserve healthspan and extend lifespan in diverse model organisms (Colman et al., 2014; Gribble & Welch, 2013; Lin et al., 2002; McCay et al., 1935; Weindruch et al., 1986). The hallmarks of CR in mammals include decreased mortality and also decreases in all major diseases of aging, including cancer, cardiovascular disease, kidney disease, diabetes, and neurodegenerative diseases (Green et al., 2022). There has been significant interest in understanding the physiological and molecular mechanisms engaged by CR that are responsible for its beneficial effects. We have recently reviewed these in depth (Green et al., 2022), but will briefly discuss a few of the key potential mechanisms that have been examined here.

Calorie restriction has been proposed to work in part due to reduced production of reactive oxygen species; however, extensive studies in genetically engineered rodents suggest that reactive oxygen species likely do not play a major role in normal aging (Salmon et al., 2010). CR substantially reduces the risk of cancer in mice; however, while the loss of transcription factor NRF2 (nuclear factor erythroid 2‐related factor) blocks the effects of CR on cancer protection, it does not block the ability of CR to extend lifespan (Pearson et al., 2008). While CR improves insulin sensitivity in all mammals, this improvement is dispensable for the benefits of a CR diet on frailty and longevity in mice (Yu et al., 2019). CR may promote health through reduced activation of specific signal transduction pathways such as PI3K/AKT (Mercken et al., 2013) and possibly mechanistic target of rapamycin (mTOR) (Bjedov et al., 2010; Unnikrishnan et al., 2020), but this remains to be tested rigorously in mammals. We recently showed that many of the benefits of CR require prolonged fasting between meals, which is necessary for CR‐induced improvements in insulin sensitivity, frailty, cognition, and longevity in mice (Pak et al., 2021).

In short, there are still many questions about the mechanisms by which CR functions to promote healthy aging. Further, the translatability of CR to humans is generally thought to be low, as most people are unlikely to be able to maintain lifelong adherence to such an abstemious diet. Thus, there is substantial interest in identifying alternative dietary regimens that will mimic the beneficial effects of CR without restricting calories.

## 3 DIETARY PROTEIN IN HEALTHSPAN AND AGING

Calorie restriction proportionally decreases the consumption of all three macronutrients (fat, carbohydrate, and protein), and for many years, the contributions of restricting these individual macronutrients to the effects of CR have been explored. It is now generally believed that the restriction of protein in a CR diet is not great enough to fully explain the benefits of CR (Speakman et al., 2016). However, restriction of protein reproducibly extends the lifespan of flies (Bruce et al., 2013; Grandison et al., 2009; Lee et al., 2008; Mair et al., 2005) and rodents (Solon‐Biet et al., 2014; Weindruch et al., 1986). Many studies have shown PR improves metabolic parameters in rodents, such as glucose tolerance, insulin sensitivity, circulating triglycerides, and other blood lipids (Fontana et al., 2016; Maida et al., 2016; Solon‐Biet et al., 2014, 2015).

These data in model organisms go against trending dietary advice for humans, which has generally recommended that humans should be eating more protein to improve satiety and promote weight loss (Cuenca‐Sanchez et al., 2015; Yu et al., 2020). High protein diets are indeed indicated for certain clinical conditions or life stages, such as pregnancy and old age, but epidemiological evidence suggests that overconsumption of protein outside of these conditions could be deleterious (Delimaris, 2013). A randomized controlled trial (RCT) of overfeeding in metabolically healthy individuals with low, normal, or high protein content found that low protein feeding resulted in significantly less weight gain, though this was a result of lack of lean mass gain rather than reduced fat gain (Bray et al., 2012). In middle‐aged overweight males, a RCT of protein restriction (PR) (feeding of a 7%–9% protein diet without calorie restriction) for 6 weeks resulted in significant body weight and fat mass loss, as well as improvements in body mass index (BMI) (Fontana et al., 2016). As evidence regarding appropriate distribution of dietary protein, carbohydrate, and fat continues to develop, the US and Canadian Dietary Reference Intake Steering Committee are planning to re‐investigate the Dietary Reference Intake (DRI) recommendations for energy and the macronutrients (*Dietary Guidelines for Americans*, *2020*–*2025*, 2020).

In summary, PR is an attractive regimen as it recapitulates many of the beneficial phenotypes of CR without requiring reduced calorie intake. Determining the mechanisms by which PR improves health is of great interest, as these can inform more specific dietary recommendations and drug targets to promote health and longevity.

## 4 THE BRANCHED‐CHAIN AMINO ACIDS

Decades ago, it was found that protein source can influence longevity; rats fed a soy protein‐based diet lived 15% longer than rats fed a comparable casein‐based diet (Iwasaki et al., 1988). One potential explanation for these different outcomes is that different protein sources have distinct amino acid profiles. For example, vegans are believed to naturally consume less methionine than meat eaters with a different balance of amino acids overall, though this idea was challenged in a recent study (MacArthur et al., 2021; McCarty et al., 2009; Schmidt et al., 2016). Methionine is a sulfur‐containing essential amino acid with roles in methylation that was first observed to extend lifespan in rats in the 1990s (Orentreich et al., 1993; Richie et al., 1994), and its lifespan extending effects have since been replicated in other models (Johnson & Johnson, 2014; Lees et al., 2017; Miller et al., 2005). A fuller discussion of the role of dietary methionine in healthy aging can be found elsewhere (e.g., Brown‐Borg & Buffenstein, 2017), but the work on methionine has highlighted the possibility that the specific amino acid composition of the diet may play a critical role in metabolic health and longevity.

The branched‐chain amino acids leucine, isoleucine, and valine are three of the nine amino acids that are known to be essential for nonruminant mammals, including mice and humans. The BCAAs are abundant in high protein foods, making up approximately 20% of the amino acids found in meat, fish, eggs, and nuts. BCAAs are hydrophobic and serve important roles at the molecular level in protein folding, substrate binding, lipid solubility, and interaction with nonpolar substrates; these amino acids are also common in coiled‐coiled α helices, which occur often in the proteins myosin, keratin, and some transcription factors (Brosnan & Brosnan, 2006).

The BCAAs are strong agonists of the amino acid‐sensitive kinase mTOR complex 1 (mTORC1). mTORC1 regulates a wide variety of downstream biological processes—most notably those related to growth and proliferation such as ribosomal, protein, nucleotide, and lipid synthesis through integration of nutrient and hormonal cues; this has been reviewed in great detail elsewhere (Babygirija & Lamming, 2021; Kennedy & Lamming, 2016). Put simply, amino acids promote the lysosomal localization of mTORC1. Binding of the BCAAs, especially leucine, to Sestrin2 relieves the inhibitory action of Sestrin2 on the GATOR2 complex, allowing the Rag GTPases to bind to mTORC1 and recruit it to the lysosome (Chantranupong et al., 2014; Wolfson et al., 2016). Several other molecular mechanisms by which BCAAs, especially leucine, regulate mTORC1 recruitment by the Rag GTPases have been discovered (Han et al., 2012; He et al., 2018; Zhu et al., 2021). At the lysosome, mTORC1’s kinase activity is allosterically activated by the binding of Rheb‐GTP (Yang et al., 2017).

The other amino acid sensor that is involved in BCAA metabolism is GCN2 (general control nonderepressible 2). Unlike mTORC1, GCN2 senses amino acid deprivation by binding to uncharged transfer RNA molecules (tRNA) and stalled ribosomes (Dong et al., 2000; Harding et al., 2019; Wek et al., 1995), and works to repress general translation and prioritize preferential production of ATF4, a transcription factor that upregulates genes necessary to adapt to PR including the energy balance hormone fibroblast growth factor 21 (FGF21) (De Sousa‐Coelho et al., 2012). Mice lacking GCN2 have a delayed metabolic response to PR, including a 2‐week delay in the induction of FGF21 (Laeger et al., 2016).

Downstream of GCN2 and ATF4 is FGF21, which is secreted from the liver and other tissues in response to nutrient stress (Nishimura et al., 2000). FGF21 is induced by PR in rodents and humans, and has been described as a key regulator of the response to PR, increasing insulin sensitivity and energy expenditure (Fontana et al., 2016; Laeger et al., 2014). Recent studies show that FGF21 signaling in the brain is required to alter food intake and increase energy expenditure during protein restriction (Hill et al., 2017, 2019), and transgenic overexpression of FGF21 has been found to extend lifespan (Zhang et al., 2012). Discussion of the role of FGF21 in response to total and specific restriction of BCAAs will be discussed in greater detail below.

## 5 BCAA CATABOLISM

After consumption, BCAAs are absorbed in the intestine by classic Na^+^‐dependent co‐transporters, and transported across other membranes by the essential amino acid antiporter L‐type large neutral amino acid transporter 1 (LAT1) (Scalise et al., 2018). While many BCAAs are utilized as building blocks for protein synthesis, BCAAs in excess of those needed for protein translation are catabolized. The first catabolic step is reversible: deamination by branched‐chain aminotransferase (BCAT), which catalyzes any BCAA and α‐ketoglutarate to a branched‐chain keto acid (BCKA) and glutamate, respectively (Harper et al., 1984). BCAT is expressed in mitochondrial and cytoplasmic isoforms, BCATm and BCATc (Hutson et al., 1992), though most BCAA catabolism takes place in the mitochondria, so intermediates can quickly enter the TCA cycle. Nearly all subsequent intermediates downstream of the BCKAs remain trapped in the mitochondria due to their conjugation to CoA, a process that is described in greater detail later (Neinast et al., 2019). BCATc is highly expressed in the brain and various CNS cells; this provides leucine‐derived nitrogen needed for the production of the neurotransmitter glutamate (Castellano et al., 2007; Hutson et al., 1998; Yudkoff, 1997).

**FIGURE 1:**
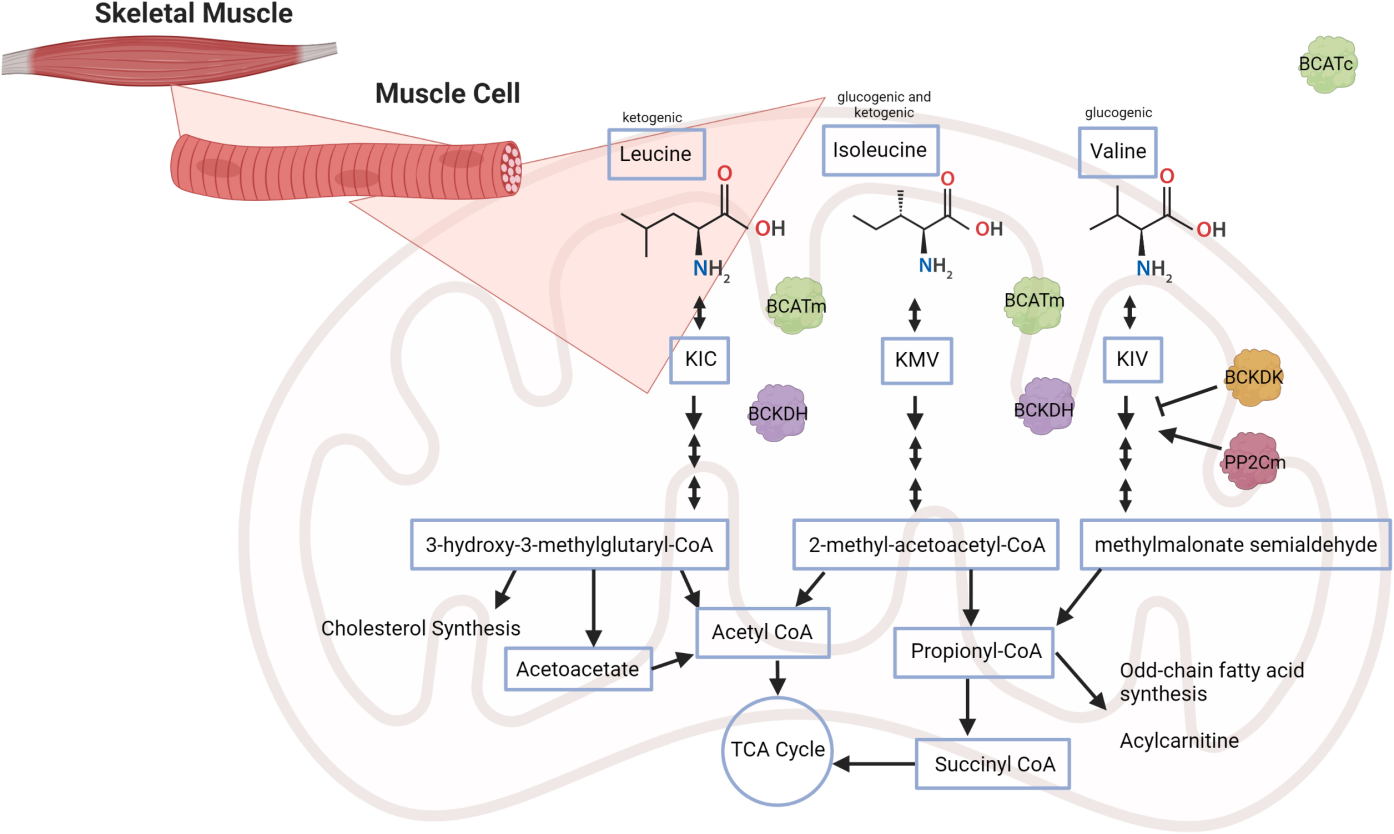
BCAA catabolism. Simplified outline of the process of BCAA catabolism into their respective ketogenic or glucogenic substrates by the skeletal muscle, though the first catabolic step, deamination, can also occur in many other tissues (but not in liver hepatocytes). KIC, α‐ketoisocaproate; KIV, α‐ketoisovalerate; KMV, α‐keto‐methylvalerate

As shown in Figure 1, BCAT catabolizes each BCAA to its respective keto acid: leucine to α‐ketoisocaproate (KIC), isoleucine to α‐keto‐methylvalerate (KMV), and valine to α‐ketoisovalerate (KIV) (Harper et al., 1984). The next step in BCAA catabolism, which is irreversible and rate‐limiting, is performed by the branched‐chain keto acid dehydrogenase (BCKDH) complex, a member of the mitochondrial α‐keto acid dehydrogenase complex family (Matthews et al., 1981; Shimomura et al., 2001). BCKDH is comprised of multiple copies of three subunits: BCKA decarboxylase (E1), dihydrolipoamide acyltransferase (E2), and dihydrolipoamide dehydrogenase (E3). BCKDH converts KIC to isovaleryl‐CoA (IV‐CoA), KMV to α‐methylbutyryl‐CoA (MB‐CoA), and KIV to isobutyryl‐CoA (IB‐CoA). BCKDH is expressed in all tissues, including hepatocytes.

BCKDH is regulated by a kinase and phosphatase and is inhibited by serine phosphorylation of the E1 subunit by BCKD kinase (BCKDK) (Shimomura et al., 1990). Conversely, dephosphorylation of BCKDH is performed by the mitochondrial protein phosphatase 1K (PPM1K or PP2Cm) (Lu et al., 2009). BCKDK and PPM1K link BCKDH activity to feeding; PPM1K is highly expressed in the fed state but lowered by fasting, while the reverse is true for BCKDK. The overexpression of the transcription factor ChREBPβ upregulates hepatic BCKDK and downregulates PPM1K expression (White et al., 2018). BCKDK is also allosterically inhibited by BCKA excess (Shimomura et al., 2001), and PPM1K is inhibited by interaction with E2 of BCKDH (Dong et al., 2013).

Following processing by BCKDH, BCAA catabolism undergoes multiple additional steps, some of which are specific to the catabolism of a specific BCAA and others of which are shared between catabolic pathways. A key point is that with the exception of 3‐hydroxyisobutyrate (3‐HIB)—a valine‐specific catabolic intermediate—the intermediates of BCAA catabolism are conjugated to CoA and thus confined to the mitochondria (Jang et al., 2016). The final products are specific to each individual BCAA: Ketogenic leucine is catabolized to acetoacetate and acetyl‐CoA, glucogenic valine to propionyl‐CoA, and isoleucine to both acetyl‐CoA and propionyl‐CoA. Propionyl‐CoA can enter the TCA cycle via conversion to succinate.

Physiologically, BCAA catabolism is partitioned to tissues for different purposes. While the liver is a major metabolic hub for most amino acid metabolism, BCATm is not expressed in hepatocytes (Suryawan et al., 1998; Sweatt et al., 2004). In contrast, skeletal muscle is both a consumer of BCAAs for protein synthesis and expresses both BCAT and BCKDH. As a result, the conventional model for amino acid catabolism has for many years suggested that although other amino acids are catabolized in the liver, BCAAs are primarily catabolized in skeletal muscle (Suryawan et al., 1998). More recent work has shown that adipose tissue is also a key player in BCAA catabolism as transplanting wild‐type adipose tissue into whole‐body *Bcatm* ^−/−^ mice is sufficient to decrease circulating BCAAs by 30%–50% (Herman et al., 2010). BCAAs are also used to fuel thermogenesis in brown adipose tissue, and BCAA transport into BAT mitochondria is essential to raise body temperature after cold exposure (Yoneshiro et al., 2019).

A recent model of whole‐body BCAA fates reconciled these recent findings with early models (Neinast et al., 2019). BCAA turnover is quite rapid once BCAAs enter the bloodstream, with transamination products visible after only 3 min, and end‐catabolism into TCA products detectable within 3–5 min. While a majority of BCAA catabolism takes place in skeletal muscle, 19% occurred in brown adipose tissue, with other tissues representing about 20% of flux. This new model verifies that even in more nuanced tracing experiments, muscle is the main tissue of BCAA catabolism, while also confirming new data on the catabolic capacity of adipose tissues. This understanding of the “normal” catabolic process aids our understanding—and the potential for therapeutic modulation—of dysregulated BCAA catabolism in insulin resistance.

### 5.1 Modulating BCAA catabolism

Genetic and pharmacological manipulation of BCAA catabolism has been used to investigate how BCAAs regulate metabolic health. Whole‐body deletion of BCKDK not only activates BCAA catabolism to promote new steady‐state and tissue levels of BCAAs and BCKAs but also impairs growth and neurological function (Joshi et al., 2006; Neinast et al., 2019). Conversely in lean mice, the whole‐body deletion of *Ppm1k* decreases but does not completely blunt BCAA catabolism, and actually improves insulin sensitivity, glucose tolerance, and weight (Lu et al., 2009). Additionally, the deletion of *Bcatm* provides resistance to diet‐induced obesity and promotes leanness and improved glucose tolerance (She, Reid et al., 2007), though these mice need access to lower BCAA diets to avoid toxicity.

Resistance to diet‐induced obesity was also replicated in a recent study of adipose‐ and iWAT‐specific *Bcatm* knockout mice (Ma et al., 2022). The deletion of *Bcatm* in these tissues improved glucose tolerance and insulin resistance, and reduced circulating cholesterol, triglyceride, and free fatty acid levels. Mechanistically, this was found to be a result of increased thermogenesis and adipose tissue browning. Further study found that this was the result of PR/SET domain 16 (PRDM16) acetylation by BCAA‐derived acetyl‐CoA, resulting in decreased binding of PRDM16 to peroxisome proliferator‐activated receptor γ (PPARγ). This leads to the suppression of browning genes and therefore contributes to diet‐induced obesity. Interestingly, this same study identified that telmisartan, an FDA‐approved antihypertensive medication, inhibits BCATm, increasing iWAT browning and energy expenditure, and reducing adiposity in mice.

Acute overexpression of *Ppm1k* in liver of Zucker fatty rats, which are obese, hyperphagic, and hyperinsulinemic, lowers hepatic triglycerides and improves glycemia, likely through action on ATP citrate lyase (ACL), rather than BCKDH, as described above (White et al., 2018). However, a greater focus has been placed on modifying BCKDK activity, as increasing BCAA disposal is logically a potential therapy for treating insulin‐resistant obesity. The compound 3,6‐dichlorobenzo[b]thiophene‐2‐carboxylic acid (BT2) is a recently identified allosteric inhibitor of BCKDK (Tso et al., 2014). In Zucker fatty rats, BT2 rapidly lowers hepatic triglycerides and improves glucose tolerance and insulin sensitivity (White et al., 2018), and treatment of *ob*/*ob* and diet‐induced obese mice with BT2 restores BCAA catabolism and is sufficient to improve glucose tolerance and insulin resistance (Zhou et al., 2019). Quantitative tracing experiments show that BT2 treatment robustly increases BCAA oxidation in skeletal muscle, though it alters phosphorylation of BCKDH in liver and heart as well (Neinast et al., 2019).

Indeed, one of the most characterized applications of BT2 is to prevent BCAA accumulation in the heart and improve cardiac function. In the heart, high levels of glucose negatively regulate BCAA catabolic enzymes through inhibition of the transcription factor Krüppel‐like factor 15 (KLF15), and BCAA accumulation along with high glucose levels produces insulin‐resistant cardiac tissue (Fillmore et al., 2018; Shao et al., 2018; Sun et al., 2016). This increases vulnerability to ischemic injury, accelerates oxidative stress and superoxide production, and has been linked to multiple types of heart failure in humans and mice (Li et al., 2017; Sun et al., 2016; Uddin et al., 2019; Wang et al., 2016). BT2 preserves cardiac function and prevents vulnerability to acute ischemia in mouse models of heart failure (Li et al., 2017; Sun et al., 2016), and can even restore cardiac function to hearts with preexisting dysfunction (Chen, Gao et al., 2019). Additional studies are warranted to find the specific mechanisms that allow improved cardiac function by BT2.

BCAA catabolism is also necessary for and altered by endurance exercise. Disruption of BCAA catabolism by *Bcatm* deletion in skeletal muscle impairs exercise performance and endurance (She et al., 2010). Conversely, mice genetically predisposed to high endurance catabolize BCAAs faster, and more efficiently; this is likely driven by increased PGC1α activation (Overmyer et al., 2015). When overexpressed in skeletal muscle, PGC1α drives BCAT and BCKD expression (Hatazawa et al., 2014), and dramatically increases the BCAA catabolic capabilities of muscle (Jang et al., 2016; Neinast et al., 2019). Clearly, perturbations in BCAA catabolism alter physiological metabolism and may be a therapeutic target in insulin resistance and tissue‐specific metabolic dysfunction.

## 6 BCAAs ARE ASSOCIATED WITH INSULIN‐RESISTANT OBESITY

Over 50 years ago, it was discovered that plasma levels of BCAAs are positively correlated with insulin resistance and obesity in humans (Felig et al., 1969). This has been expanded upon in more recent studies of obese and insulin‐resistant humans around the world (Chen, Akter et al., 2019; Huffman et al., 2009; Newgard et al., 2009; Xu et al., 2013), as well as in laboratory models of diabetes and obesity (Lynch & Adams, 2014; She, Van Horn et al., 2007). High BCAA levels can also be predictive of diabetes onset (Wang et al., 2011), especially in post‐renal transplant recipients (Oste et al., 2020). This prognostic association has also been observed in adolescents (McCormack et al., 2013). More recently, elevated BCAAs have been associated with negative cardiovascular outcomes (Du et al., 2018; Le Couteur et al., 2020; Portero et al., 2021; Sun et al., 2017).

Normal BCAA levels can be restored through weight loss; a comparison of plasma amino acid levels in over 1000 individuals from two randomized dietary weight loss trials found that pounds of weight lost correlated well with decreased BCAAs (Zheng et al., 2016). Other studies have found that plasma BCAA levels during weight loss interventions are correlated with improvements in glucose homeostasis and insulin sensitivity (Laferrere et al., 2011; Shah et al., 2012). This correlative effect between BCAAs and metabolic parameters has raised a question among researchers: Are BCAAs pathogenic in insulin‐resistant obesity, or a by‐product of accelerating metabolic syndrome?

To answer this question, several groups have combined data from genome‐wide association studies (GWAS) with BCAA and insulin levels. One meta‐analysis found several single nucleotide polymorphisms (SNPs) in genomic regions of BCAA catabolism and connected these SNPs to elevated BCAAs and insulin resistance (Lotta et al., 2016). Two similar studies presented opposing results, postulating that though BCAAs are elevated in type II diabetes, this is caused by genetic risk for insulin resistance (Mahendran et al., 2017; Wang et al., 2017). Recent research in this field has concentrated on what increases circulating BCAAs, how this interacts with dysregulated metabolic states such as insulin resistance, and what methods are most effective at restoring BCAA levels.

Another recently published GWAS utilized the generation of a genetic risk score (GRS) of five common BCAA metabolic pathway SNPs in conjunction with dietary BCAA intake to parse out determinants of T2DM risk. This study was conducted in a Chinese population of almost 10,000 individuals and determined that a high GRS plus high intake of BCAAs conveyed the greatest risk for T2DM development. Interestingly, high BCAA intake positively correlated with HbA1c and circulating BCAA levels in participants with a high but not a low GRS (Wang et al., 2021). In a cohort of over 1600 Mexican adults, rare SNPs in both BCATm and BCKDH genes were associated with elevated body weight and BMI, fasting blood glucose, and blood pressure, compared to their peers with more common alleles (Vargas‐Morales et al., 2021). Individuals with the rare variants also had higher amounts of isoleucine, methionine, proline, and aspartate in circulation, though follow‐up studies are needed to determine how this impacts overall health. Together, these data suggest that a personalized nutrition approach with specific attention to the BCAAs might be warranted, particularly in those with a high genetic risk for diabetes development or with certain SNPs in the BCAA catabolism pathway.

BCAA catabolism is altered by insulin resistance, and this likely perpetuates a cycle where elevated BCAAs promote further insulin resistance and keeps BCAAs elevated. This is exemplified in adipose tissue, where BCAA catabolic enzymes decrease in activity and expression in insulin‐resistant WAT (Lackey et al., 2013; She, Van Horn et al., 2007). Furthermore, disrupting BCAA catabolism or preventing BCAA transport in BAT is sufficient to lower body temperature and accelerate insulin‐resistant obese phenotypes in mice (Yoneshiro et al., 2019). This was confirmed in tracing experiments of two insulin‐resistant mouse models, where catabolism was shifted away from adipose tissues, with increased reliance on skeletal muscle and heart (Neinast et al., 2019).

In both humans and rodents with insulin‐resistant obesity, elevated BCAAs are associated with decreased levels of glycine, particularly in muscle tissue (Newgard et al., 2009; White et al., 2016). It is postulated that glycine is depleted in an attempt to clear lipid metabolites that accumulate in muscle as a hallmark of insulin resistance. However, lipotoxicity is further exacerbated by the increased requirement for BCAA catabolism in muscle. Recently, a catabolite of valine, 3‐hydroxyisobutyrate (3‐HIB), was discovered to promote fatty acid trans‐endothelial uptake into skeletal muscle, providing a key and previously unknown link between BCAA catabolism and fatty acid transport in muscle (Jang et al., 2016); this catabolite is also associated with an increased future incidence of insulin‐resistant obesity, even after adjustment for BCAAs (Mardinoglu et al., 2018). By this mechanism, increased dependence on skeletal muscle for BCAA catabolism only furthers insulin resistance.

The liver is another site of dysregulated metabolism in insulin resistance; though liver represents <10% of BCAA oxidation, this tissue represents 27% of BCAA disposal into protein synthesis (Neinast et al., 2019). Elevated hepatic BCAAs prevent GCN2‐mediated repression of SREBP1c and fatty acid synthase (FAS), which drive lipogenesis (Guo & Cavener, 2007). Additionally, the BCKDH regulators BCKDK and PPM1K also target ACL in the liver, another key enzyme in lipogenesis, and in insulin‐resistant conditions, high BCKDK and low PPM1K inhibit BCKDH while promoting ACL activity (White et al., 2018). By these mechanisms, elevated BCAAs can increase insulin resistance by promoting hepatic lipogenesis in inappropriate conditions. Indeed, diets with reduced levels of total BCAAs, isoleucine, or valine reversed hepatic lipid accumulation in Western diet‐induced obese mice, even as the mice continued to consume an otherwise high‐fat, high‐sucrose Western diet (Cummings et al., 2018; Yu et al., 2021). As the liver, muscle, and adipose tissues all alter BCAA catabolism in insulin‐resistant states, some researchers have explored altering these catabolic pathways in an attempt to treat these conditions and learn more about the pathogenicity of elevated BCAAs.

## 7 BCAA DISPOSAL IN PROTEIN AND BCAA RESTRICTION

The field of BCAA metabolism has been advanced with the use of large, multi‐omics data analyses and metabolic tracing experiments that can determine the tissue‐specific use of AAs. The new model proposed by Neinast et al. is certainly a more comprehensive assessment of whole‐body BCAA catabolism (Neinast et al., 2019); however, there are still a few outstanding questions. Although this updated model seems to agree with previous literature, it only accounts for approximately 50% of whole‐body BCAA disposal; for example, one major metabolic organ not evaluated was the gut, home to intestinal microbiota that can produce BCAAs and other essential amino acids (Lynch & Pedersen, 2016; Pedersen et al., 2016; Ridaura et al., 2013). Furthermore, there are discrepancies in the literature regarding the expression and modification of BCAA catabolic enzymes, and how this relates to tissue‐specific BCAA oxidation. It will be interesting to see how models of BCAA catabolism evolve, especially as delineation of sexual dimorphisms is prioritized in research.

In insulin‐resistant conditions, BCAA catabolism is decreased in adipose tissue and shifted toward muscle. However, it is unknown how BCAA disposal and oxidation change in protein and BCAA restriction. When fewer BCAAs are fed, BCAA incorporation into protein would presumably proportionally increase, oxidation would decrease, and the distribution of BCAA uptake would also likely change. BCKA oxidation is elevated in liver of Zucker lean rats and elevated in muscle of Zucker fatty rats (White et al., 2016), which agrees with a model that BCAA oxidation is shifted toward muscle in insulin‐resistant conditions.

## 8 DIETARY INTAKE OF BCAAs AFFECTS HEALTH AND LONGEVITY

As early as the 1980s, scientists reported that modifying the BCAA content of diet can regulate activity of catabolic enzymes (Block et al., 1985). Dietary BCAA intake is associated with increased weight and adiposity in both aged male mice and humans (Ribeiro et al., 2019), and decreasing BCAA consumption through PR was reduced blood levels of BCAAs in two human trials (Fontana et al., 2016; Maida et al., 2017). In a large nutritional geometry diet composition study, circulating BCAAs were the only amino acids (AAs) that correlated with dietary protein intake in mice (Solon‐Biet et al., 2014). The use of AA‐defined diets has allowed specific modification of BCAA intake in several rodent and human trials during the last decade.

### 8.1 BCAA supplementation in rodents

Though it may seem paradoxical to the literature presented so far, there have been several instances where BCAA supplementation has been reported to improve health. Specific supplementation of leucine in drinking water prevented hyperglycemia and decreased weight and fat gain from high‐fat diet feeding in mice, not by decreasing energy intake but by increasing resting energy expenditure and UCP3 (uncoupling protein 3) expression in adipose and muscle (Zhang et al., 2007). Additionally, supplementation of BCAAs (plus 8 other amino acids) in drinking water to mice from 9 months of age onward slightly increased lifespan in a single study; this extension was attributed to increased mitochondrial biogenesis, decreased reactive oxygen species, and improved exercise capacity (D'Antona et al., 2010).

However, more recent and carefully controlled studies have reported that BCAA supplementation, particularly in the context of a Western diet, increases glucose intolerance and insulin resistance in rodents (Cummings et al., 2018; Newgard et al., 2009). In rats, this supplementation also increased muscle mTORC1 activity, and BCAA‐induced insulin resistance was acutely reversed by rapamycin treatment (Newgard et al., 2009). Another recent study found that doubling dietary BCAAs promoted hyperphagia, obesity, and early mortality (Solon‐Biet et al., 2019). A time‐of‐day feeding study also identified that feeding BCAA‐enriched meals at the end of wake periods in mice resulted in enhanced cardiovascular growth and detrimental remodeling in a circadian clock‐dependent manner (Latimer et al., 2021). These studies differ in means and degree of supplementation, which may explain some of these seemingly contradictory results.

### 8.2 BCAA restriction promotes metabolic health in rodents

Both BCAA restriction and deprivation have been studied in rodents; as BCAAs are essential, deprivation experiments are only sustainable short‐term. Initial deprivation experiments examined the effects of feeding mice leucine‐free, isoleucine‐free, or valine‐free diets for up to a week. These deprivation regimens rapidly improved glycemic control and liver insulin sensitivity (Xiao et al., 2011, 2014). These diets appeared to influence canonical pathways of PR, as all three diets were associated with decreased mTORC1 and increased AMPK activity in the liver. Activation of mTORC1 signaling via S6K1 or whole‐body deletion of *Gcn2* was sufficient to reduce benefits of BCAA deprivation on insulin sensitivity. In valine‐deprived diet conditions, at least some of these effects appear to be mediated by GCN2, as whole‐body *Gcn2* ^−/−^ mice were slightly less insulin‐sensitive than wild‐type mice when fed a valine‐free diet.

More recent studies have focused on the more physiologically relevant reduction in dietary BCAAs. These studies typically examined a 50%–80% restriction of BCAAs, which in contrast to complete removal of one of these essential amino acids, is sustainable over the entire lifespan. A 67% restriction of all three BCAAs initiated at 9 weeks of age in male mice—approximately equivalent to a human teenager (Flurkey et al., 2007)—improves metabolic health and recapitulates many effects of decreasing consumption of PR (Fontana et al., 2016; Yu et al., 2021). These mice weighed less despite increased food intake, primarily as a result of increased energy expenditure and reduced fat mass accretion. BCAA restriction also improved glucose and pyruvate tolerance equivalently to a PR diet.

As BCAA restriction was extremely successful in promoting metabolic health without negative side effects and could be fed for sustained periods of time in mice, we and others have tested the effects of restricting dietary BCAAs on diet‐induced obese mice and other diabetic rodent models. In agreement with the hypothesis that elevated BCAAs contribute to insulin resistance by increasing muscle lipotoxicity, a study using a 45% restricted BCAA diet completely normalized accumulation of fatty acyl‐CoAs and restored skeletal muscle insulin sensitivity in 6‐week‐old Zucker fatty rats to levels found in lean rats (White et al., 2016). BCAA restriction also improves fatty acid oxidation and triglyceride levels in hearts of Zucker fatty rats and shifts fuel selection from glucose to fatty acid catabolism (McGarrah et al., 2020).

Mice eating a Western diet were transitioned to Western diets in which the three BCAAs or all amino acids were restricted by 67% at 18 weeks of age, approximately equivalent to a human in their middle twenties (Flurkey et al., 2007). These mice rapidly returned to a normal body composition, losing the adipose mass and weight gained during the previous 12 weeks of Western diet feeding in about 4 weeks. They also demonstrated substantial improvements in glucose tolerance and insulin sensitivity. Thus, decreasing BCAA consumption is potent enough to counteract an otherwise unhealthy Western diet and rescue a metabolically unhealthy mouse (Cummings et al., 2018).

### 8.3 BCAA restriction promotes fitness and longevity in mice

As a BCAA‐restricted diet is quite effective and pervasive in improving metabolic health, and recapitulates many of the effects of a PR diet, and BCAAs are agonists of mTOR signaling, researchers have investigated the effects of a BCAA‐restricted diet on longevity. Consistent with the negative effect of BCAAs on longevity, dietary supplementation with extra BCAAs leads not only to impaired metabolic health but also to decreased lifespan (Cummings et al., 2018; Mu et al., 2018; Newgard et al., 2009; Solon‐Biet et al., 2019). Mice fed a 50% or 80% restricted BCAA diet from 12 weeks of age did not live longer (Solon‐Biet et al., 2019); similarly, a 67% BCAA‐restricted diet improved the metabolic health and reduced the frailty of male and female mice when started at 16 months of age, but did not increase lifespan (Richardson et al., 2021).

However, we have found that lifelong restriction of BCAAs by 67% extends the longevity of two short‐lived mouse models of Hutchinson–Gilford progeria syndrome (Richardson et al., 2021). In wild‐type mice, the dietary restriction of BCAAs by 67% initiated at weaning reduces frailty and extends the lifespan of male, but not female, mice by over 30%. These animals displayed reduced mTORC1 signaling in multiple tissues, specifically in males (Richardson et al., 2021). In combination, these studies suggest that the precise level of restriction, time of diet initiation, and sex may play a role in determining whether BCAA restriction will extend lifespan. Further, while the sex‐specific effects of BCAA restriction on mTORC1 signaling may explain the male‐specific benefits of BCAA restriction on lifespan, the effect of BCAAs on mTORC1 activity is likely dispensable for the effects of reduced BCAA diets on metabolic health.

### 8.4 Dietary BCAAs in human health and longevity

In humans, acute BCAA supplementation has been extensively studied as a way for athletes and the elderly to build or preserve muscle mass. BCAA supplementation before and after exercise promotes muscle protein synthesis and decreases exercise‐induced muscle damage in humans (Howatson et al., 2012; Shimomura et al., 2004). In mice, BCAA supplementation improves body composition when wheel access and exercise is allowed (Platt et al., 2016), indicating that BCAA supplementation may benefit those who are regularly exercising. As more studies focus on circadian biology and nutrient timing, it will be interesting to see whether BCAA supplementation yields improvements or disadvantages based on the time of administration even in nonexercising models. Elevated BCAAs are specifically associated with poor health outcomes in humans overall, and higher blood levels of isoleucine are associated with increased mortality, while in humans, higher dietary levels of isoleucine are associated with body mass index (Deelen et al., 2019; Yu et al., 2021). However in the elderly, especially the frail elderly, BCAAs are decreased (Adachi et al., 2018; Chaleckis et al., 2016; Ottestad et al., 2018; Ter Borg et al., 2019). Similarly, in the elderly increased protein or essential amino acid supplementation improves frailty outcomes (Dillon et al., 2009), blood glucose control (Solerte et al., 2008), and lean mass (Solerte et al., 2008).

In addition to frailty, the risk of dementia, related neurological complications, and neurodegenerative diseases increases with age. Blood levels of BCAAs are elevated in the blood of humans with Alzheimer’s disease (AD) and mouse models of the disease, and brain BCAA catabolism is impaired in the brains of models of AD by downregulation of *Bcat1* expression (Li, Ye et al., 2018). Dietary supplementation of BCAAs leads to increased cognitive deficits and increased phosphorylation of Tau, and, when combined with a high‐fat diet, leads to the premature death in the 3xTg mouse model of AD (Tournissac et al., 2018). Dietary restriction of BCAAs instead improves the cognitive performance of 3xTg AD mice (Tournissac et al., 2018). It is likely that increasing BCAA consumption may improve health outcomes or quality of life in some specific conditions or life stages, and future research should try to determine what factors predispose humans to respond positively to BCAA supplementation.

### 8.5 BCAA restriction as a clinical intervention

There are many factors that need to be considered in the application of experimental diets to human patients, mainly safety and feasibility. BCAA‐restricted diets could conceptually be used as a weight loss and insulin‐sensitizing intervention or promote healthy longevity. Restricting BCAAs in diet‐induced obese mice induced rapid weight loss (Cummings et al., 2018), and this dramatic effect would likely not be tolerated well or sustained in humans. Though mice of both sexes tolerate lifelong BCAA restriction well, some females who began the diet in midlife suffered early mortality, which is an obvious safety concern. However, young mice and diet‐induced obese mice do not adversely react to a diet restricted by two‐thirds BCAAs, and this appears to be a safe dietary intervention if weight is carefully monitored.

Clinical experiments utilizing dietary BCAA restriction in humans are sparse; to date, there are two trials using this dietary regimen. The first study was conducted in metabolically healthy individuals and utilized whole foods in addition to medical‐grade foods and formulas that are engineered for individuals with maple syrup urine disease (MSUD). MSUD is an autosomal recessive disease in which mutations in BCKDH genes require limitation of the BCAAs to prevent neurotoxic buildup of BCAAs and BCKAs. Over the course of a week, the intentional reduction in dietary BCAAs resulted in reduced circulating BCAAs by 50%. Additionally, the intervention slightly reduced insulin resistance as measured by Homeostatic Model Assessment of Insulin Resistance (HOMA‐IR) (Ramzan et al., 2020). Presumably, a longer intervention would yield significant improvements in insulin resistance and overall glucose homeostasis, but more clinical trials are needed, especially in populations with underlying metabolic conditions.

In a second clinical trial of BCAA restriction, BCAAs were reduced by supplementing subjects eating a low protein diet with either a complete AA mixture or one lacking BCAAs for one month. BCAA‐restricted humans demonstrated lowered circulating BCAAs in several feeding states, especially during a mixed‐meal tolerance test, and improved some measures of metabolic health. Postprandial insulin secretion was lowered, oral glucose sensitivity was improved, and FGF21 increased by 21% (Karusheva et al., 2019). These exciting results prove efficacy and feasibility of decreasing BCAA consumption in humans, at least for a short period of time.

**FIGURE 2:**
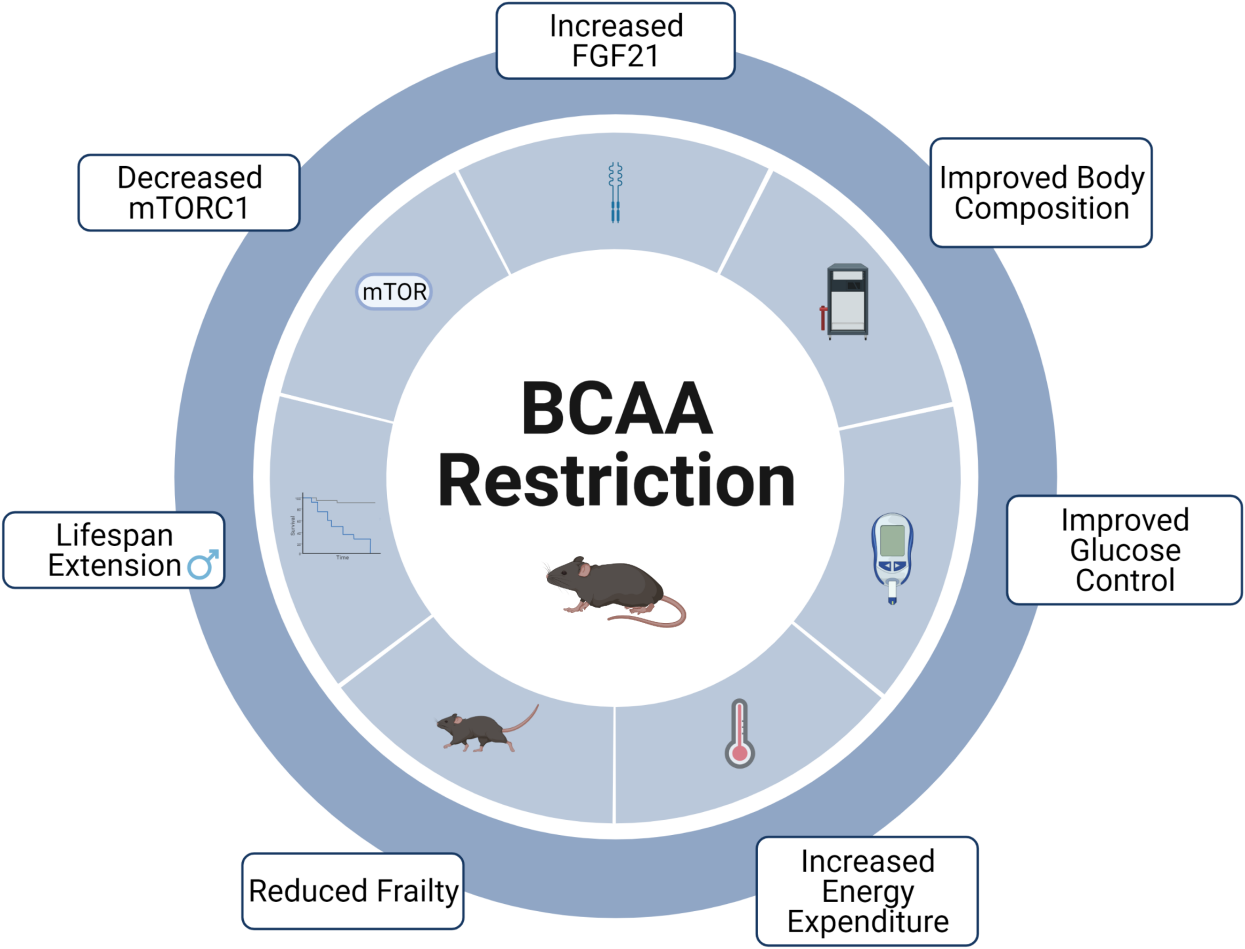
BCAA restriction improves health and extends lifespan. Visual summary of the effects of restricting dietary BCAAs on molecular signaling, healthspan, and longevity

However, a recent study of mice feeding periodized PR found that many benefits were transient and reversed after PR ended, and it is unknown whether this will apply to humans on a protein or BCAA‐restricted diet (Li, Rasmussen et al., 2018). Future studies should extend BCAA‐restricted periods, and track metabolic parameters after the diet ends, to see how interventions such as these affect long‐term health. Other solutions could involve registered dietitian‐designed diets that naturally control BCAA intake. The outcomes of dietary BCAA restriction are summarized in Figure 2.

## 9 DISTINCT METABOLIC EFFECTS OF THE INDIVIDUAL BCAAs

While BCAAs are commonly grouped together and referred to collectively, there is evidence that these AAs can have individual effects, as alluded to earlier in this review. To test the effects of individual BCAA restriction on metabolic health, mice were fed a new series of individually restricted BCAA diets. Restricting leucine instead of all BCAAs does not improve metabolic health—rather, it slightly increases adiposity (Fontana et al., 2016; Yu et al., 2021). Interestingly, valine and isoleucine restriction both improved metabolic health. Specific valine deprivation has also been shown to reduce leukemic burden and increase survival in mice with acute lymphoblastic leukemia (ALL) by reducing expression of valine tRNAs that are typically upregulated in this condition (Thandapani et al., 2022). Furthermore, adding isoleucine or valine back to a diet with low levels of all other AAs markedly reduced the benefits to metabolic health. Overall, while reduction of isoleucine and valine proved to drive the benefits of a BCAA‐restricted diet, and leucine often had no effect when manipulated in diet alone, isoleucine restriction elicited the most potent improvements to metabolic health in all conditions tested. Through the use of genetically altered mice, it was determined that isoleucine restriction improves metabolic health independently of hepatic mTORC1 and GCN2, and that FGF21 may play a role in these benefits (Yu et al., 2021). Overall, these data show that while BCAAs are often referred to collectively and grouped together in analysis, they have distinct physiological roles.

It is curious that the individual BCAAs do not produce equal effects when restricted. The catabolism of these AAs is regulated by the same kinases, though their end products differ by glucogenic and ketogenic properties. In a standard chow diet, leucine is the most abundantly fed BCAA, and isoleucine is the least abundant, so it is tempting to think that when restricted by 67%, isoleucine and valine have crossed some threshold that results in improved metabolic health. Indeed, when leucine is restricted by 80%–85%, there are benefits to metabolic health, and FGF21 was only induced after 85% leucine restriction (Lees et al., 2017; Wanders et al., 2015). However, even in experiments that eliminate individual BCAAs, differences among their effects were still observed. Xiao et al. showed that though eliminating any individual BCAA rapidly reduces weight and improves insulin sensitivity, only valine and isoleucine elimination improved glucose tolerance and lowered fed blood glucose levels, possibly through decreased expression of key gluconeogenic genes (Xiao et al., 2011, 2014). Furthermore, one study of methylmalonyl‐CoA mutase heterozygosis in mice, which prevents complete valine and isoleucine oxidation to succinyl‐CoA, resulted in susceptibility to insulin‐resistant obesity (Lerin et al., 2016). However, the distinctions among the BCAAs that influence metabolic heath are still unclear, and future work should determine whether and how diets limited in leucine, isoleucine, and valine are differently sensed.

Finally, as isoleucine restriction produces such potent effects compared with the other BCAAs, and as BCAA restriction extends longevity, feeding an isoleucine‐restricted diet and testing the effects on longevity is a logical next step. Though isoleucine is the BCAA most influential in improving metabolic health, metabolic health is not always linked to increased lifespan. Several groups have now shown that insulin sensitivity and longevity can be uncoupled (Arriola Apelo et al., 2020; Lamming et al., 2012; Selman et al., 2008; Yu et al., 2019) however, BCAA‐restricted mice of both sexes are also less frail with age. It will be interesting to determine whether isoleucine restriction recapitulates these benefits on frailty and longevity.

## 10 SEXUAL DIMORPHISM IN RESPONSE TO PROTEIN AND BCAA RESTRICTION

Sex differences in longevity have been observed in almost every species studied, but there is a clear lack of research evenly into both sexes (Austad & Fischer, 2016; Zucker & Beery, 2010). Modern longevity studies have found substantial effects of sex on lifespan and disease burden (Le Couteur et al., 2018; Mitchell et al., 2016), so it is perhaps unsurprising that sexually dimorphic effects have also been found in mice subject to protein and BCAA restriction.

As previously discussed, consumption of a 67% BCAA‐restricted diet starting in midlife improved metabolic health and reduced frailty in both sexes, though this diet only extended lifespan in males when initiated at a young age (Richardson et al., 2021). Intriguingly, this was also the case with respect to PR; a PR diet started early in lifespan only extended the lifespan of males (Richardson et al., 2021). The only other study evaluating BCAA restriction and longevity to date did not find any difference in either sex with longevity, and found most effects were not dimorphic, though this study used a more strict level of BCAA restriction than ours (Solon‐Biet et al., 2019). Some insights into these results can be drawn from research into dietary protein intake.

A recent publication tested how different levels of dietary protein impacted the metabolic health and molecular profile of multiple strains of male and female mice. While some phenotypes were conserved across strains and sexes, including increased glucose tolerance and energy expenditure, there was large variability in adiposity, insulin sensitivity, and circulating hormones with sex, strain, and age of onset (Green et al., 2022). This study also demonstrated that short‐term PR was effective at improving metabolic health when started much later in life, and the pattern of sexual dimorphism was altered at old age.

Another experiment testing different protein:carbohydrate ratios in both sexes on reproduction and lifespan found that female mice maximize reproductive health and longevity at different ratios compared with male mice. Male longevity was optimized at lower ratios than females, indicating that the degree of PR increasing male lifespan is more severe than in females. Additionally, reproductive function is maximized on higher ratios in both sexes, meaning that higher protein intake is needed for optimal reproductive health (Solon‐Biet et al., 2015). This has also been demonstrated in *Drosophila melanogaster*, as females suppress egg production on low‐protein, high‐carbohydrate diets (Lee, 2015).

These results are useful when thinking about BCAA restriction, as it is conceivable that to increase lifespan in females, different levels of limitation may be needed. At the two‐thirds level of restriction tested, females did not have diminished mTORC1 activity (Richardson et al., 2021). Future experiments could test different degrees of BCAA restriction and measure how mTORC1 signaling and lifespan respond in female mice. Furthermore, effects on reproductive health may differentially influence female vs. male longevity, and it would be interesting to determine how BCAA restriction influences reproductive health in both sexes.

## 11 FUTURE DIRECTIONS IN BCAA RESEARCH METABOLISM AND AGING

### 11.1 The role of FGF21

**FIGURE 3:**
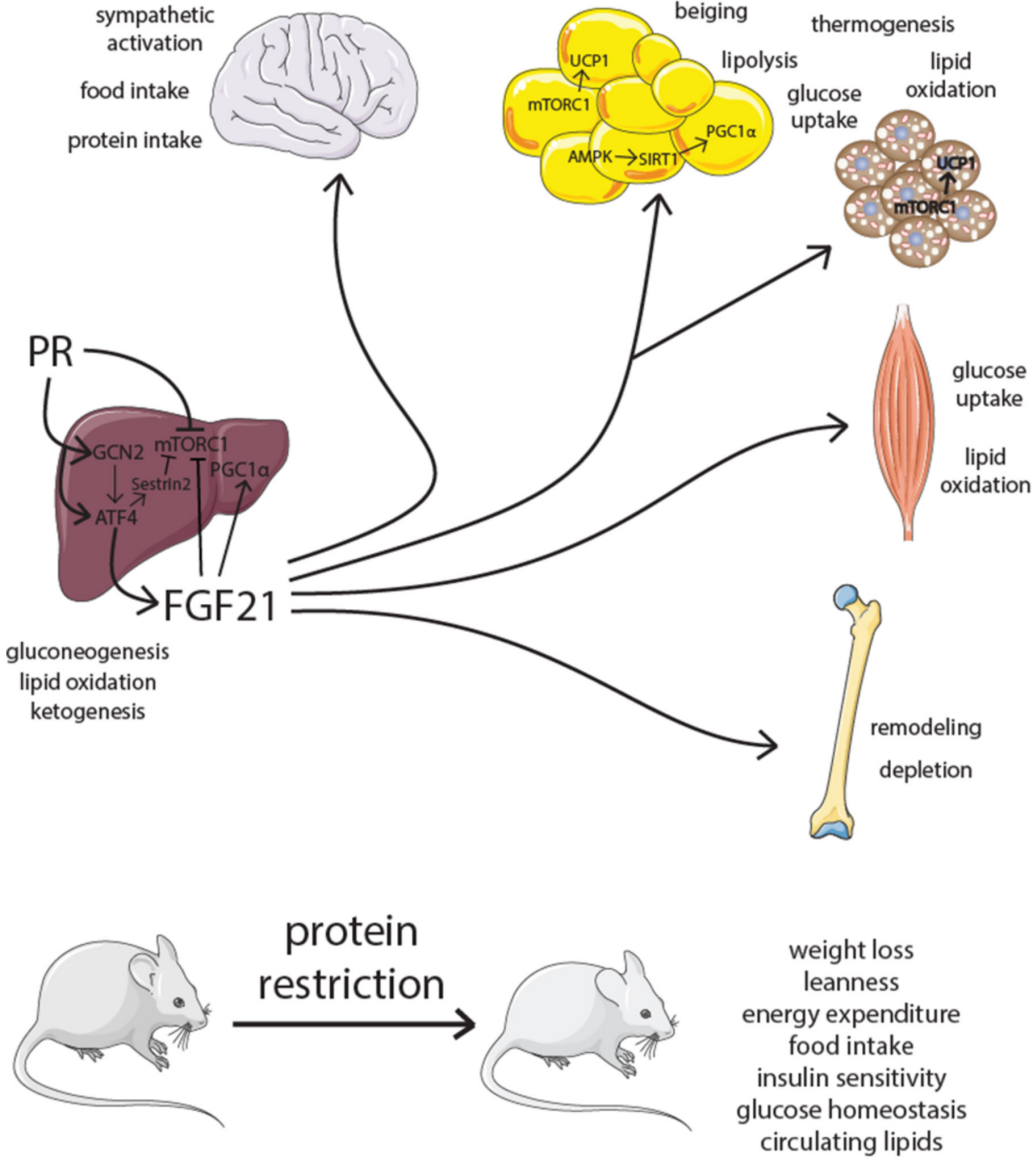
FGF21 in PR. PR induces hepatic FGF21 resulting in metabolic and physiological effects in many tissues. Note: FGF21 also acts through autocrine and paracrine means not illustrated here

Probably the largest remaining question in the work presented so far is the role of FGF21 in response to BCAA or isoleucine restriction. As FGF21 is essential for the metabolic benefits and lifespan extension of PR (Hill et al., 2022; Laeger et al., 2014) (Figure 3), and as BCAA and isoleucine addback to a PR diet blunts or eliminates these metabolic benefits (Yu et al., 2021), it is logical to hypothesize that FGF21 is essential for the effects we observe in BCAA and isoleucine restriction. Several studies have examined the effect of BCAA restriction on FGF21 levels in mice. Two studies found that BCAA restriction did not increase blood levels of FGF21 (Fontana et al., 2016; Solon‐Biet et al., 2019), while a third observed increased FGF21 expression in aged males fed a Low BCAA diet (Richardson et al., 2021). Another study observed that BCAA restriction study in the context of a Western diet temporarily increased FGF21 (Cummings et al., 2018). Two studies of BCAA repletion in PR have presented divided results, with one reporting that BCAA addback did not blunt increases in FGF21 by PR (Maida et al., 2017), and the other showing a slight decrease (Mu et al., 2018).

The role of FGF21 in response to specific restriction of isoleucine is a bit clearer; isoleucine restriction strongly raises FGF21 levels and induces *Fgf21* transcription in multiple tissues. Moreover, deletion of *Fgf21* blocks isoleucine‐induced increases in food consumption and energy expenditure. However, *Fgf21* ^−/−^ mice on a low isoleucine diet still displayed improved glucose tolerance and body composition (Yu et al., 2021). Modifying hepatic AA sensing by deletion of *Gcn2* or *Tsc1* (Tuberous sclerosis complex 1—an upstream negative regulator of mTORC1) does not alter the metabolic response to isoleucine restriction (Yu et al., 2021). Another study has shown that hepatic ATF4 can activate and increase FGF21 expression without GCN2 activity, though this process is delayed (Laeger et al., 2016). The molecular mechanisms by which FGF21 levels are increased by isoleucine restriction, and the molecular processes by which dietary isoleucine restriction promotes glucose tolerance and reduces adiposity, remain to be determined.

Interestingly, our laboratory recently discovered that the metabolic and molecular response to PR has sex‐ and strain‐dependent effects (Green et al., 2022). Though many benefits of PR diets are attributed to FGF21, these studies have primarily been conducted in C57BL/6J males. Surprisingly, we observed that while blood levels of FGF21 are strongly induced by PR in C57BL/6J males, this increase was more muted in C57BL/6J females, and not observed in DBA/2J or HET3 mice of either sex. Further, these results uncoupled increased energy expenditure on PR diets and metabolic health improvements, and in DBA females, EE negatively correlated with hepatic *Fgf21*. These results suggest that many PR outcomes may be independent of changes in FGF21.

## 12 CONCLUSIONS

There are still questions surrounding dietary AAs, metabolism, and longevity that remain unanswered by the current literature, especially in humans. The work discussed above raises new questions about how the amount and quality of protein intake influences health, and suggests that perhaps these dietary recommendations will need to be personalized. For example, as detailed earlier, the elderly may need to consume more BCAAs to prevent muscle loss and stave off frailty, while athletes may need to consume more BCAAs to build and maintain muscle. These protein or BCAA recommendations may be personalized based on one’s circulating amino acid levels and genes, allowing us to find the best diet for each person. Further research into the molecular mechanisms which underlie the benefits of BCAA and protein restriction may allow the development of pharmaceuticals to mimic these dietary interventions.

## 13 CONFLICT OF INETERST

D.W.L has received funding from, and is a scientific advisory board member of, Aeovian Pharmaceuticals, which seeks to develop novel, selective mTOR inhibitors for the treatment of various diseases.

## AUTHOR CONTRIBUTIONS

All of the authors contributed to all aspects of the article.
